# New Technologies in Cereal Processing and Their Impact on the Physical Properties of Cereal Foods

**DOI:** 10.3390/foods12214008

**Published:** 2023-11-02

**Authors:** Min Li, Meng Niu

**Affiliations:** College of Food Science and Technology, Huazhong Agricultural University, Wuhan 430070, China; li_min@webmail.hzau.edu.cn

Cereal is a general term for cereal plants or food crops, covering a wide range of foods, including rice, wheat, millet, corn and other miscellaneous grains, and representiing the most important component of the human diet. Cereals are rich in many types of nutrients required by humans, including proteins, starches, lipids, trace elements, and vitamins, and are the main source of energy for the body. Cereals play a key role in the diet as staple foods. An adequate intake of cereals can regulate bodily mechanisms and reduce the incidence of diseases such as hypertension, diabetes, and vascular diseases. In recent years, the consumer demand for quality grains has been growing higher and higher, with the aim of not only retaining more nutrients but also reducing the number of processing steps. This has meant that many diverse new processing technologies have emerged. New cereal processing technologies not only increase the grain yield and protect grain nutrients, but also help to improve the main end-product texture and sensory quality and other functional characteristics. The physical properties of foods are closely related to the type of processing technology employed, and appropriate processing technologies can produce cereal food products with different attributes. There are key technical challenges facing traditional cereal processing, such as nutrient loss and underutilization, which constrain the development and use of cereal foods (for example, whole-wheat steamed bread or brown rice). Therefore, more and more advanced processing technologies are needed. This Special Issue is dedicated to a collection of studies on new cereal processing technologies and their effects on the physical properties of food. These papers aid us in understanding the connections between cereal processing technology and the physical properties of food from multiple perspectives, on both the micro- and macro-levels, and lay the foundation for the development of new cereal foods with desirable organoleptic and textural attributes.

Steam explosion (SE) is a new technology for safer and more efficient grain processing that has developed rapidly in recent years. After the raw material is heated to 180~235 °C with steam, the pressure is maintained for a period of time and then suddenly relieved to produce a second release of steam to make the volume surge. It can break the connections within lignocellulose, which not only promotes the release of the active ingredients in food materials but also modifies macromolecules to enhance the corresponding functional properties. In the study by Dang et al. [1], steam-exploded wholegrain highland barley powder showed a good swelling power, water-holding capacity, and oil-holding properties with a minimum bulk density; thus, it could be used as the raw processing material for high-oiliness baking products. SE also provided a better storage stability for wholegrain flours. Kong et al. [2] reported that SE treatment improved protein digestibility, starch digestibility, and phenolic bioaccessibility in wholemeal flour. The chemical changes in wholemeal flour induced by steam explosion caused alternations in the flour’s solvent-retention capacity and rheological properties, and altered the falling number (and liquefaction number). Liu et al. [3] provide evidence that 0.8 MPa is the optimal condition for SE pretreatment for maximizing the nutritional and functional proprieties of wheat bran. They added this wheat bran to wheat flour to study the changes in textural and sensory qualities. The addition of SE bran (0.8 MPa) reduced the peak viscosity, final viscosity, and setback and increased the pasting temperature of the flour–bran mixtures. The addition of 6% SE black-grained wheat bran (0.8 MPa) to chiffon cakes led to a low hardness and chewiness, a high antioxidant activity, and an excellent total score. The study by Hong et al. [4] shows that under optimum conditions, which comprise a steam pressure of 2.34 MPa, a processing time of 37.0 s, and an initial water content of 10.0%, steam expansion significantly improves the structure, hydration, and swelling performance of qingke barley, while retaining its nutritional characteristics and antioxidant properties. 

High-hydrostatic-pressure (HHP) technology typically uses water as the pressure transfer medium and adopts a pressure of 100–1000 MPa to treat materials to achieve the goals of sterilization, changes in enzyme activity, quality improvement, and so on, and to meet the demand for freshness and for less processing. HHP is widely used in food (grain and legume) processing, especially in the modification of wholegrain, flour, and legume proteins, and in the improvement of the quality of pasta and legume products. For example, Lin et al. [5] explored the effects of HHP (150, 300, 450, and 600 MPa for 5, 10, or 15 min) processing on the rheological, pasting, thermal, and functional properties of bean flours. They found that HHP enhanced the G′, G″, and gel strength of common bean flour according to the increase in the pressure and holding time, and this may be caused by the increase in molecular interactions and the strengthening of the microstructure. Shorstkii et al. [6] show how HHP treatment accelerated the soaking process of wheat grains and thus positively affected their pasting characteristics. Seo et al. [7] investigated the effect of HHP treatment on the physicochemical properties of rice flour with different moisture contents. The results show that the water-absorption capacity, solubility, and swelling power of rice flour, according to the moisture content and HHP treatment, tended to increase with increases in the moisture content and pressure, and that the HHP-treated rice flour had smaller starch granules and fewer voids. Zhu and Li [8] reported that HHP at 500 and 600 MPa decreased the viscosity during pasting, the gel hardness, the enthalpy change (ΔH) in gelatinization, and the in vitro starch digestibility of quinoa flour, while increasing the water solubility.

High-pressure homogenization (HPH) is a processing technology that changes the average particle size and microstructure of a material through violent collisions between the material and the homogenizing valve at high speeds. During this process, the properties of the material, such as the adsorption and rheology, are also changed. HPH has many advantages over conventional grinding processes. Examples include a smaller particle size, a larger surface area and viscosity, and a greater oil-holding capacity, cation exchange, and water-holding and swelling capacity. These improved properties not only improve the rheological properties of the dough/batter but also extend the shelf life of baked goods and improve the color, texture, and sensory properties of the food. Sert et al. [9] found that bread made from HPH-treated flour mash had a more attractive brightness, a lower hardness, and an improved cohesion and resilience compared to the controls. Li et al. [10] treated corn starch with HPH and found that HPH treatment significantly increased the transmittance of starch pastes (from 7.6% to 81.3%) (*p* < 0.05) and that the average molecular size of corn starch was negatively correlated with the HPH pressure. It was also noted that the content of starch chains with a degree of polymerization (DP) of 37–100 and a DP of 2000–20,000 decreased significantly (*p* < 0.05) with the increasing intensity of HPH treatment. A significant (*p* < 0.05) increase in the viscosity of non-glutinous rice was observed when the rice was cooked with HPH-treated starch paste solutions, and 60 MPa and a 3% starch concentration were determined to be the optimal conditions for HPH treatment to increase the rice viscosity. Tang et al. [11] found that HPH treatment not only significantly increased the content of straight-chain starch in rice starch but also formed a more stable starch crystalline structure and a short-range ordered structure. In addition, the slurry produced via HPH treatment has a medium viscosity, which is more favorable for the formation of steamed rice bread with a larger specific volume, a better texture, and a lower starch digestibility. Zhao et al. [12] discovered that HPH altered the static rheological characteristics of quinoa protein by decreasing the shear stress and viscosity. It also modified the thermal stabilities of quinoa protein by increasing its thermal denaturation temperature.

Pulsed electric field (PEF) processing has emerged in the last two decades and is a non-thermal processing technique in which short pulses are applied to the sample material by means of a high voltage applied between two electrodes. Liquid samples offer an ideal processing medium for PEF because of their electrical conductivity; however, in recent years, it has been found that PEF can also change the structural properties of solid materials. Qiu et al. [13] investigated the physicochemical properties, microstructure, and pasting behavior of PEF-treated and non-PEF-treated glutinous rice grains. The researchers treated glutinous rice with different strengths of PEF at a field strength of 3 kv/cm, and the results showed that the porosity of the surface of the rice grains increased, and the peak viscosity of the glutinous rice flour decreased significantly, after PEF treatment. Qiu et al. [14] further investigated the effects of PEF treatment on the storage properties of rice gel (GR-Gs, made from glutinous rice grains and gum) and rice cakes (GR-Cs, made from glutinous rice grains, sugar, oil, and gum). During storage, the hardness of GR-Gs and GR-Cs increased significantly, while the adhesion values decreased. In addition to its use in processing rice-based foods, PEF can also be used to modify gluten in wheat. Zhang et al. [15] explored the changes in gluten properties that came with adjusting the PEF parameters. The results showed that the total free sulfhydryl groups in gluten increased significantly, and the total sulfhydryl groups decreased significantly, with increasing electric field strength, resulting in a significant decrease in the hydrophobicity of the gluten surface. Hong et al. [16] found that adding PEF-assisted esterified starch, especially PEF-assisted B-type starch, was beneficial in improving the hardness of noodles and increasing the stretching distance. These results shed new light on the role of PEF technology in the texture and quality of cereal products.

Plasma, as the fourth state of matter, is primarily composed of photons, ions, and free electrons, as well as atoms in their fundamental or excited states with a net neutral charge. The methods used for the generation of plasma includes dielectric barrier discharges, corona glow discharges, radio frequency, and gliding arc discharges. In addition to the mode of generation, the type of excitation gas is also an important factor affecting the results of treatment with plasma. Plasma may be generated via the ionization of many gases, for instance, O_2_, H_2_, He, Ne, Ar, NH_3_, CH_4_, or CF_4_. Plasma, as a green technology to change material characteristics, has attracted much attention in cereal processing in recent years. Liu et al. [17] studied the changes in the physicochemical properties of oats at different plasma treatment times (0, 5, 10, 15, and 20 min at an output discharge voltage of 60 kV). They indicated that low-temperature plasma treatment improved the water-holding capacity, swelling power, and solubility, and reduced the peak viscosity, final viscosity, regeneration value, and enthalpy of oat flour. In addition, it could suppress the deterioration of oat flour. Chaple et al. [18] investigated the effect of atmospheric cold plasma on the physicochemical and functional properties of wheat flour and found that a series of treatment times (5–30 min) in a dielectric barrier discharge plasma reactor at 80 kV for whole-wheat grains and wheat flour improved the hydration properties of the flour and increased the final viscosity of the paste, the enthalpy of heat absorption, and the degree of crystallinity. Zhou et al. [19] found that the pH of waxy maize starch and normal maize starch was reduced after treatment with 5% starch suspension (*w*/*w*) and an atmospheric pressure plasma jet (APPJ) for a short period of time (1, 3, 5, or 7 min). In addition, APPJ treatment significantly increased the water-binding capacity of starch and decreased the relative crystallinities, pasting temperatures, and enthalpies of pasting. In the study by Lokeswari et al. [20], atmospheric-pressure cold plasma treatment on pearl millet (*Pennisetum glaucum*) with air as the feed gas, at an input voltage of 40 and 45 kV for 5, 10, and 15 min exposure times, was studied. The results indicated that hydration properties, such as the swelling capacity, solubility, and water absorption showed a significant increase with the treatment time and voltage. Moreover, the pasting properties, such as the peak, trough, break down, set back, and final viscosity increased with the exposure time and voltage.

## References

[1] Dang B., Zhang W.-G., Zhang J., Yang X.-J., Xu H.-D. (2022). Effect of Thermal Treatment on the Internal Structure, Physicochemical Properties and Storage Stability of Whole Grain Highland Barley Flour. Foods.

[2] Kong F., Zeng Q., Li Y., Zhao Y., Guo X. (2022). Improving bioaccessibility and physicochemical property of blue-grained wholemeal flour by steam explosion. Front. Nutr..

[3] Liu Y., Huang S., Meng T., Wang Y., Zhang Z. (2023). Effects of steam explosion on the nutritional and functional properties of black-grained wheat bran and its application. J. Sci. Food Agric..

[4] Hong Q., Chen G., Wang Z., Chen X., Shi Y., Chen Q., Kan J. (2020). Impact of processing parameters on physicochemical properties and biological activities of Qingke (highland hull-less barley) treated by steam explosion. J. Food Process. Preserv..

[5] Lin T., Fernández-Fraguas C. (2020). Effect of thermal and high-pressure processing on the thermo-rheological and functional properties of common bean (*Phaseolus vulgaris* L.) flours. LWT.

[6] Shorstkii I., Sosnin M., Mounassar E.H.A., Bindrich U., Heinz V., Aganovic K. (2022). The Effect of High-Pressure Pre-Soaking on the Water Absorption, Gelatinization Properties, and Microstructural Properties of Wheat Grains. AgriEngineering.

[7] Seo J.-H., Jo Y.-J., Lee Y.-R., Lee J., Jeong H.-S. (2023). Physicochemical Properties of Soft and Hard-type Rice Flour According to Moisture Content and High Hydrostatic Pressure Treatment. Foods.

[8] Zhu F., Li H. (2019). Effect of high hydrostatic pressure on physicochemical properties of quinoa flour. LWT.

[9] Sert D., Mercan E., Tongur A., Kara Ü. (2021). Production of bread from doughs composed of high-pressure homogenisation treated flour slurries: Effects on physicochemical, crumb grain and textural characteristics. J. Food Meas. Charact..

[10] Li H., Yan S., Yang L., Xu M., Ji J., Liu Y., Wang J., Sun B. (2020). High-pressure homogenization thinned starch paste and its application in improving the stickiness of cooked non-glutinous rice. LWT.

[11] Tang Z., Fan J., Yang J., Liu L., He L., Zhang W., Zeng X., Qin L. (2022). Rheological, texture and in vitro digestibility properties on steamed rice bread modified by ultrafine grinding and high pressure homogenization on rice-okara slurry. Int. J. Food Sci. Technol..

[12] Zhao Y., Yuan Y., Yuan X., Zhao S., Kang Z., Zhu M., He H., Ma H. (2023). Physicochemical, conformational and functional changes of quinoa protein affected by high-pressure homogenization. LWT.

[13] Qiu S., Abbaspourrad A., Padilla-Zakour O.I. (2021). Changes in the Glutinous Rice Grain and Physicochemical Properties of Its Starch upon Moderate Treatment with Pulsed Electric Field. Foods.

[14] Qiu S., Abbaspourrad A., Padilla-Zakour O.I. (2022). Prevention of the Retrogradation of Glutinous Rice Gel and Sweetened Glutinous Rice Cake Utilizing Pulsed Electric Field during Refrigerated Storage. Foods.

[15] Zhang C., Yang Y.-H., Zhao X.-D., Zhang L., Li Q., Wu C., Ding X., Qian J.-Y. (2021). Assessment of impact of pulsed electric field on functional, rheological and structural properties of vital wheat gluten. LWT.

[16] Hong J., Li C., An D., Liu C., Li L., Han Z., Zeng X.-A., Zheng X., Cai M. (2020). Differences in the rheological properties of esterified total, A-type, and B-type wheat starches and their effects on the quality of noodles. J. Food Process. Preserv..

[17] Liu S., He T., Rafique H., Zou L., Hu X. (2022). Effect of low-temperature plasma treatment on the microbial inactivation and physicochemical properties of the oat grain. Cereal Chem..

[18] Chaple S., Sarangapani C., Jones J., Carey E., Causeret L., Genson A., Duffy B., Bourke P. (2020). Effect of atmospheric cold plasma on the functional properties of whole wheat (*Triticum aestivum* L.) grain and wheat flour. Innov. Food Sci. Emerg. Technol..

[19] Zhou Y., Yan Y., Shi M., Liu Y. (2018). Effect of an Atmospheric Pressure Plasma Jet on the Structure and Physicochemical Properties of Waxy and Normal Maize Starch. Polymers.

[20] Lokeswari R., Sharanyakanth P.S., Jaspin S., Mahendran R. (2021). Cold Plasma Effects on Changes in Physical, Nutritional, Hydration, and Pasting Properties of Pearl Millet (*Pennisetum glaucum*). IEEE Trans. Plasma Sci..

